# Why flipping the classroom is not enough: Digital curriculum making after the pandemic

**DOI:** 10.1007/s11125-021-09555-9

**Published:** 2021-04-28

**Authors:** Susanne Backes, Isabell Baumann, Dominic Harion, Sabrina Sattler, Thomas Lenz

**Affiliations:** Maison des Sciences Humaines, University of Luxembourg, 11, Porte des Sciences, 4366 Esch-sur-Alzette, Luxembourg

**Keywords:** Covid-19, Digital curriculum, Digital learning, Digital teaching, Homeschooling

## Abstract

To slow down the proliferation of Covid-19, governments virtually shut down public life, temporarily closed schools, and forced teaching to be done exclusively on a remote basis. These measures offer an opportunity to reexamine conventional teaching and learning arrangements, test new digital and analogue concepts, and provide essential inspiration for curriculum making in the twenty-first century. This article addresses the historical development of schooling in the classroom as differentiated from “homeschooling”. On one hand, the question of how school closures and digitally supported teaching settings may affect an increase in educational inequalities is investigated using an international comparison. On the other hand, the pedagogical and didactical implications of distance learning and a digital teaching culture, which constitute the foundation for digital curriculum making, are examined.

To slow the proliferation of Covid-19, governments virtually shut down public life, temporarily closed schools, and forced teaching to be done exclusively on a remote basis. UNESCO (2020) estimated that, as of March 23, 2020, school closures around the world affected 90.2% of students. One obvious intervention was to switch to digitally supported teaching models; these, however, were implemented very differently and to varying degrees, depending on infrastructural equipment and curricular adaptability. As diverse and differentiated as the initiatives have been nationally and internationally, they also offer the opportunity to rethink conventional teaching and learning arrangements, explore new digital and analogue concepts, and provide essential stimuli for curriculum making in the 21st century. Thus, the curriculum response to the Covid-19 crisis becomes a beacon for the development of a digital culture in education systems, which are also facing pre-pandemic concerns that go hand in hand with an increasingly heterogenous society. Among these concerns are socioeconomic factors, professional mobility, nationality, and linguistic background.

The small country of Luxembourg, where 60% of the student population is composed of non-Luxembourgish students, offers a nearly ideal laboratory setting for examining the processes relevant to virtually and digitally enhanced classroom instruction in multilingual and intercultural contexts during the coronavirus crisis. Luxembourg’s centralized education system is characterized by a separation between primary and secondary schools, with compulsory schooling from age 4 (including one year of preschool) to age 16. After grade 6, students are directed toward one of three main secondary school tracks. One result is that Luxembourg’s secondary school system is highly stratified (Backes 2020). School tracks end with different school-leaving certificates and differ in terms of students’ competencies. Substantial disparities regarding placement and competencies exist between students from different socioeconomic backgrounds (Lenz and Heinz 2018). Further key characteristics are Luxembourg’s trilingual education system (with Luxembourgish, German, and French as languages of instruction) and its highly heterogeneous student population (42.5% of students non-Luxembourgish in 2016–2017; UNESCO 2020). What can the Luxembourgish laboratory tell us about the future of digital curriculum making?

To better understand the processes relevant to digitally enhanced classroom instruction in multilingual and intercultural contexts during the coronavirus crisis, we will first sketch the historical lines of development of the nationalization of schools, as differentiated from “homeschooling” (section 2). We will look at how school closures and digitally supported teaching settings may increase educational inequalities (section 3), as reflected in an international comparison (section 4). We will also consider the pedagogical and didactical implications of distance learning and a digital teaching culture, which constitute the foundation for digital (in contrast with digitally supported) curriculum making (section 5).

## The state and the schools

Schools are—and this applies, of course, not just to Luxembourg—institutions capable of cultivating a feeling of national belonging, so it is no accident that the modern school system emerged in historical parallel to the modern nation-state (Baumann 2019). The educational researchers Ramirez and Ventresca (1992) summed up the relationship between the individual, the state, and the school as an institution as follows: “Mass schooling becomes the central set of activities through which the reciprocal links between individuals and nation-states are forged” (p. 24). This close association between school and nation was in no way a given and is new in both historical and comparative terms. In the ancient world, children usually learned through observation and imitation at home, and only wealthy families could afford a tutor. In the Middle Ages, schooling became a task of the church; monastery schools provided for the education of a numerically small future caste of priests. The beginnings of secular schooling are found in the late Middle Ages, in the 12th century, when schools emerged in larger European cities mainly for the sons of wealthy merchants, who were educated in order to follow in their fathers’ professional footsteps (Konrad 2012). From a historical perspective, then, schools developed from a private to an ecclesiastical and later to a state affair. The closeness of the links between state and school have evolved in very different degrees around the world. For example, while home-based instruction is not allowed in Germany, large church-run schools exist alongside the public school system, Luxembourg has pursued a joint state-church school policy that allows homeschooling in principle, even if parents rarely decide to take up this option. In the United States, private schools and homeschooling play a much larger role.

The US educational system grants parents the fundamental right to provide instruction at home, and parents do so with greater frequency than in European countries. Government intervention varies from state to state. This dual view of instruction, as a state and yet individual task, can be attributed to, among other things, a specifically Anglo-American understanding of curriculum as more than just the planning of instruction. In the United States, homeschooling is made possible by the historically explicable notion of the teacher and a specific kind of instruction (instructional design), which is produced in the form of curricula and can be distinguished from the continental European tradition of Didaktik.

The idea of instructional design describes systematic planning and the procedure and evaluation of learning processes, learning environments, and learning materials. With the aid of textbooks, instructional materials, and standardized tests, instruction should, in theory, be possible for anyone. This idea developed during the progressive era (1880–1920), a time in which national consciousness and national identity played a major role in social and educational reforms as well as political movements (Popkewitz 2019). Sociodemographic factors played an important role at a time when US society was confronted with increased immigration and the consequences of urbanization. Education policy reacted by strengthening the orientation of instructional organization toward “public needs” (Westbury 1995, p. 218), thereby incorporating the notion of democratic education and the idea of social progress (Tröhler 2014). In this context, teaching concepts developed that are structurally embedded within the curriculum. According to the traditional Anglo-American understanding, there is no systematic differentiation between curricular matter and lesson meaning, or between teaching and instructional planning, within a curriculum (see Hopmann 2015, p. 16).

In Germany and Luxembourg, however, curricula determine content, forms, and testing to a far lesser extent. The individual teacher is supposed to act as the mediator between content and the student. In addition, above all in Germany, there is a different, less liberal and individualistic understanding of the state, which is prepared to grant the individual fewer rights in such fundamental questions as schooling.

With the coronavirus pandemic’s contact restrictions and school closures, the state was now forced to hand over responsibility for the school sector to the private sphere, to families. Thus, out of purely epidemiological necessity, teaching reverted to the status of homeschooling and of observing, imitating, and editing standardized teaching material. This posed a number of difficulties because almost all the prerequisites for a successful transfer of school responsibility were missing, and the curricular, pedagogical, and even purely technical prerequisites for functional homeschooling did not exist.

This has medium and long-term effects on teaching practices, the fabric of teaching and learning, and our understanding and framework of education in general. Of particular importance is the question of the effects of forced homeschooling and distance learning on access to education and the possible exacerbation of social and educational inequalities. Distance learning clearly highlights and seems to aggravate the so-called *digital divide*, but it also increases the *digital use divide*, the differences in knowledge and skills for the responsible use of digital resources. Therefore, we will first take a look at the connection between digitization and educational inequalities.

## Educational inequality and barriers

The digital divide has been a subject of debate since the mid-1990s (Zillien 2009). It is therefore not surprising, especially during this pandemic, that scientists and the media have examined the connection between homeschooling or digital schooling and educational inequalities. One frequent pattern of explanation in public discourse focuses on the topic of technical infrastructure, or the digital equipment of socioeconomically disadvantaged students. From the perspective of teaching staff, the lack of digital equipment for students is the greatest challenge of homeschooling, according to the results of a survey conducted shortly after school closures in Germany (forsa 2020).

If we take one step back from the acute crisis, the first question we must address is which findings from inequality research—independent of aspects of homeschooling and digital education—identify various vulnerabilities. As part of the “modern project of a legal and welfare state”, a demand for educational justice has emerged (Fend 2009, p. 38) that, in the 20th century, became incorporated into the formulation of human rights. International bodies have adopted conventions that demand equal educational opportunities regardless of ascriptive characteristics such as class, gender, and nationality, and ban discrimination (Meyer and Ramirez 2005). Despite these achievements, international educational research has repeatedly found that socioeconomically disadvantaged people often have lower school grades, competence levels, and school-leaving certificates and more often repeat school grades or drop out of school (for Luxembourg, see Lenz and Heinz 2018). On the one hand, this could be due to a lack of material resources such as teaching materials, extra tuition, or access to private schools (“economic capital”, according to Bourdieu 1983). It may also result from the fact that some parents do not have sufficient schooling and are less able to help with learning or that parents with lower levels of education value education and training less than do parents with high educational status (cultural capital). In addition, lower-status parents are less likely to have access to social networks (social capital) they can use to support their children’s educational trajectory (e.g., when looking for an internship or a job). Moreover, even with the same level of achievement, students from disadvantaged households tend to attend schools that lead to lower qualifications, meaning that socioeconomic status exerts an influence on educational decisionmaking (Boudon 1974). Educational inequalities have an effect; for example, immigrants are often overrepresented in lower-performance school tracks in stratified education systems, even though the motivation and educational aspiration of students with a migration background are certainly very pronounced.

How do groups of students differ with regard to the digital divide? An international study on the digital literacy of 8th graders concluded that there were origin-related differences in digital literacy in all participating countries (Fraillon et al. 2019). These findings, according to recent research, also apply to primary school children (Köhn et al. 2020). According to Paus-Hasebrink and colleagues (2019), origin-related differences are to some extent due to family media socialization. As “access to technology no longer wholly determines potential inequalities”, Hargittai and Walejko (2008) preferred the term participation divide, which is based on their finding that students from higher socioeconomical backgrounds engaged in a mix of online and offline creative activities more often than did students from lower-status backgrounds. With regard to the digital equipment of adolescents, a recent special evaluation of the German International Computer and Information Literacy Study (ICILS) data for 2018 concluded that 36% of 8th graders with low parental status in their family had none or at most one of the required digital devices (compared with 15% for privileged families; Olbrisch 2020). Against this background of diagnosed differences, homeschooling, as practiced during the coronavirus crisis, risks widening existing inequalities as several unfavorable factors accumulate. It is worth taking a closer look at homeschooling, digital learning, and distance learning with regard to their impact on educational inequalities. During school closures in the pandemic, all three aspects occur together.

Whether it is freely chosen or involuntary during a pandemic, *homeschooling* means a transfer of educational responsibility to the parent(s) or caregiver. This will have different consequences, depending on the situation at home. If the parents themselves have completed a more advanced level of schooling and are familiar with the subject matter, speak the language(s) of instruction (which is particularly relevant in Luxembourg’s trilingual school system), and have the professional flexibility to support their child professionally and emotionally during homeschooling, the child will likely have a very good learning environment. However, if there is a lack of resources (e.g., fast Internet access, printers, craft and creative materials) or of a quiet workplace for studying, and if parents are less able to replace the didactic and subject expertise of the teachers, homeschooling can have a negative impact on learning progress. Among students who were already alienated from school before the school closures (Hadjar et al. 2015), these negative attitudes toward school and learning are likely to intensify in the course of the pandemic if a feeling of dependency sets in. For parents who cannot support their children to the extent required by the situation, this can create an enormously stressful situation. Moreover, homeschooling can have an impact on the family situation. There are indications that schooling at home often falls disproportionately to mothers.

In *distance learning*, as it is currently practiced, the factor that most greatly exacerbates disparities is the lack of proximity to teachers and classmates. In everyday school life, feedback, socioemotional support, peer learning, and group work all play important roles. The familiar spaces and schedules in the school, with their defined roles and rituals, are not possible to the same extent in a distance-learning arrangement (see section 5). According to the neuroscientist Joachim Bauer (2010), dispensing with the emotional component in teacher-student interactions can lead to a loss of motivation, up to and including stress symptoms: “Being seen and appreciated is a prerequisite for activating the motivation systems of the human brain” (p. 7). “Not being seen” can therefore have greater consequences for those students who have to study alone at home than for those whose parents support them. In times of distance learning, pedagogical relationships thus take on a new quality. Contact between committed teachers and students is maintained via telephone, digital (social) media, and sometimes door-to-door visits. Some students, however, cannot participate in this, as they and their parents are simply not available. Previous parental work and the school’s communication culture can also play a role here. In addition, the structural connection mentioned by Bourdieu (1984) may have its full effect here. Since the learning culture of the school and the corresponding expectations are best suited to the general constitution and cultural capital of the upper middle class, parents from lower social strata are less likely than parents of high status to take advantage of teachers’ offers of conversation, due to inhibition thresholds (Hadjar et al. 2010). Research on resilience shows how important it is for children and adolescents from socially disadvantaged families to have unrelated adults who serve as role models; this research deals, among other things, with educational successes against the odds. One finding here is that, in addition to the ambitions of students and the amount of time they spend studying and participating in extracurricular activities, supportive people, such as teachers, can play an important role as significant others and thereby compensate for intra-family cultural capital deficits (European Commission 2018; Portes et al. 2009). This is especially true for students from non-academic families facing upcoming educational or career decisions (Backes 2020, p. 156).

*Digital learning* is a coherent learning concept. In this pandemic, however, educators often resort to digital learning as a learning method. As we have noted, a digital gap exists if all students are not equally well equipped with the necessary digital devices. Moreover, the digital competence of students is distributed along an axis of inequality. According to this understanding, the level of digital literacy has an influence on expected learning success during homeschooling. Students who are already digitally literate are better able to navigate through the broad array of materials, complete teachers’ assignments, and further develop their subject-related and information and communication technology (ICT) competences (see sections 4 and 5). In contrast, digital learning can present high barriers for students who are less competent in the use of digital learning tools, regardless of whether they are very good at a subject or not. Since new learning opportunities are not equally accessible, low digital literacy can then accumulate, with further subject-related learning deficits and experiences of frustration (i.e., explained by cumulative advantage theory, according to Merton 1988). Since primary school children in times of homeschooling are more dependent on parental support, and not only with regard to purely technical access to learning materials and tasks, we can assume that the younger the school population, the greater the inequality gap during the pandemic.

It is clear that the combined situation of learning at home relying increasingly on digital tools and digital communication channels (at least in secondary education), and without direct personal contact with teachers, is challenging, especially for already disadvantaged children and adolescents. Students with special needs face further difficulties that are difficult to overcome.

Given the dimensions of inequality outlined above, that can result from homeschooling in times of a pandemic, it is best if students return to education within their learning community as soon as possible. Nevertheless, in this time of crisis, developments are emerging that could provide inspiration for future school development, including educational equity. A look outside the box is especially helpful for considering the coherent integration of digital learning worlds.

## The plurality of teaching and learning cultures, and thinking outside the box: Digital curricula and school crisis intervention in international comparison

The coronavirus shock is developing into a catalyst for digitization in schools, and no one is exempt. However, national school systems have exhibited different degrees of preparation for this new reality.

Even before the coronavirus crisis, individual project initiatives had already begun in Luxembourg, such as *Digital Classroom Luxembourg*; learning apps, such as *MathemaTIC*; and framework documents, such as the *Media Compass* (Service de Coordination de la Recherche et de l’Innovation pédagogiques et technologiques [SCRIPT] 2019), which was presented at the beginning of March 2020 and is intended to contribute to the development of computer and media skills among Luxembourg students. The digital infrastructure in schools was also under development. Since the 2017–2018 school year, secondary schools have had the opportunity to equip students with tablets as part of pedagogical projects. Currently, 32 secondary schools, with a total of 16,998 tablets, are involved in this One2One project. Each class in a secondary school that is part of One2One must therefore not only equip each of its students with a tablet but also work on a teaching program adapted to the electronic medium.

Despite these efforts, Luxembourg students performed comparatively poorly in terms of ICT and media skills before the coronavirus crisis, according to the second ICILS, in which 8th-grade students from 14 countries were tested for computer *and* information literacy (CIL) in 2018. In the CIL tests, Luxembourg students scored an average of 482 out of 700 possible points, putting them in 10th place (Fraillon et al. 2019, p. 75). Only Chile, Italy, Uruguay, and Kazakhstan scored lower. Denmark, South Korea, and Finland scored best. The test also critically examined the accuracy and usefulness of information from multiple digital sources. This is one of the key skills of the 21st century, and it has become even more important in the wake of the coronavirus crisis, as a massive spread of fake news and conspiracy theories in the social media has paralleled the spread of the virus.

In addition to CIL, computational thinking (CT) skills were also measured in ICILS. Nine countries took part in these tests, and Luxembourg scored the worst (South Korea best, ahead of Denmark and Finland; Fraillon et al. 2019, p. 103). In other educational studies, such as PISA, Denmark and Finland, together with Estonia, have been among the top performers in European comparisons for years. What do these countries do differently?

Estonia and Finland both identified the curriculum as the hub of digital innovation. In both countries, students are taught together for nine years and then take different educational paths. The school systems of Finland and Estonia, which are less stratified than those in Luxembourg, are one of the reasons both countries are among the pioneers in terms of equal opportunities. Though, in contrast with Luxembourg, Estonia is one of the significantly less prosperous EU countries, it nevertheless has an exemplary school system, especially with regard to digital learning opportunities. Estonia had already established e-learning structures by the end of the 1990s as part of the Tiger Leap government program, which invested nationwide in a future-oriented digital structure (Ruus and Resika 2017), and every school was given Internet access. Since 2018, the Ministry of Education has funded the digitization of all textbooks for every subject from the 1st to the 9th grade. This allows teachers to consult a wide range of books when selecting the most appropriate tasks and explanations for their specific student body.

In addition, since 2002, all schools have been using the digital class app eKool as their standard. During the pandemic, eKool has guaranteed ongoing school operation: the teaching process is made transparent for students, teachers, and parents alike via the digital infrastructure, and the performance level of the students can be tracked accordingly. This minimizes the danger of students “getting lost” during school closures. In general, Estonia is setting an example in school development with its ICT equipment and is making top remote learning solutions available online on the *Education Nation* platform to support school systems that are less well equipped to deal with Corona.

The current Estonian national curriculum, which has been in place since 2014, identifies digital media literacy as one of the eight basic competences firmly anchored in the national curriculum. These competences are promoted in an interdisciplinary way and thus proactively extend the traditional cultural skills of reading, writing, and arithmetic. The national curriculum sets the standards that must be implemented by all schools. These parameters define the learning objectives, learning outcomes, assessment criteria, and evaluation procedures, as well as the requirements for the learning and teaching environment, the organization of teaching, the school-leaving certificate, and the school curriculum. Each school designs its own curriculum, based on the national curriculum. A look at the national curriculum shows that a critical approach to digital media has to be an essential part of both basic school and upper school lessons.

The situation is similar with the Finns. However, they go a step further in terms of mature pedagogical concepts geared to students and technology: in the course of a major curriculum reform in 2014–2017, Finland broke up and revolutionized the canon of subjects (Halinen 2018). Inspired by pragmatist and social constructivist learning theories, which also draw on Anglo-American curriculum history, the Finnish curriculum no longer focuses solely on disciplinary knowledge of individual subjects, but rather has expanded to include transversal core competencies (Marsh, Díaz Pérez, and Escárzaga Morales 2019). Students are thereby given the opportunity to link different but interdependent (disciplinary) learning content from the core curriculum for basic education; this integrative approach also enables learners to apply their knowledge inside and outside the educational institution (Finnish National Board of Education 2016).

Open learning formats, such as phenomenon-based learning, are entirely in keeping with this multidisciplinary approach (see section 5). Here, students study an event or project in an interdisciplinary way, including the use of digitally supported technologies. Learning units from different subjects can be combined in a modular way and explored alongside the purely disciplinary content. In contrast with Luxembourg, Finland and Estonia have a more homogeneous student population, a factor that fundamentally affects the potential of such teaching models. In light of the Finnish curriculum reform, however, it is clear that digitization does not mean that digital competences are imposed on a previously analogue teaching concept. The *flipped classroom* alone is therefore not sufficient; digital learning formats must be conceived in a completely new framework concept, especially since ICT competence is seen as an important civic characteristic (Finnish National Board of Education 2016). Finland encourages independent and self-confident use of digital media at an early age, and this is firmly anchored in the curriculum: learning to type on the keyboard is part of the standard repertoire of a primary school student, and research with the help of digital tools is taught in the core curriculum for individual subjects.

Like their Estonian neighbors, the Finns also rely on a digital class register. Although equipping schools nationwide with digital resources was an important basic step, both Estonia and Finland have harmonized their curricula with regard to digital education, among other things. A key aspect of this process was that various educational actors from various levels of aggregation, including non-governmental organizations (NGOs), worked together. The core curriculum was successively reformed for several educational levels, from early childhood education to the upper secondary school branch (Halinen 2018). The curriculum design is therefore a conglomerate of a top-down and bottom-up process, since on the one hand, different interest groups were involved, and on the other hand, the core curriculum grants the schools autonomy to be able to respond to local- and community-specific needs. For school-internal curricula, the core curriculum therefore serves as a framework document, and the local educational institutions can decide autonomously to what extent they tailor their own work plans in accordance with the core curriculum. Conceptually, the core curriculum has very clear learning objectives, which is why the framework curriculum is not overloaded with content and schools can fill in the details. From this point of view, the local-specific curricula complete the general core curriculum.

In Luxembourg, those students who participated in the One2One project were, at least technically, well equipped for the remote teaching phase triggered by the pandemic. However, before the pandemic, only 18% of students interviewed stated that they used ICT devices for school purposes on a daily basis at school, and 27% did so outside school (Fraillon et al. 2019, p. 121). The *Media Compass*, which was presented by the Luxembourg Ministry of Education one week before the coronavirus-related lockdown, provides an overview of the media skills to be mastered and offers initial ideas for implementation in class. However, this document is not binding for schools, and its content is even less fixed in the curricula (see section 5). If we take a look at the current curricula of the 8th grade (i.e., the grade in which students were tested in ICILS), it quickly becomes clear that, in Luxembourg, digital media and the related competences do not have the status of a basic competence as is the case, for example, in Estonia.

In summary, compared with Luxembourg students, Finnish and Estonian students were generally well equipped for remote teaching even before the coronavirus crisis, which is obviously related to the mandatory inclusion of digital media literacy in the curriculum.

The development of such a digitally supported curriculum requires a definition of what is meant by digital cultures in school education systems and how their development transforms the pedagogical framework. In addition to understanding how schools can use digital educational standards to implement strategies for crisis intervention, for example in the context of school closures, the Covid-19 crisis could provide an important long-term impetus for future-oriented teaching development.

## Digital cultures and new models of teaching and learning

New technologies spread primarily where there is already a need for them, and crises usually create and drive such a need (Stalder 2018). In the educational system, digitality is no longer a new cultural skill, and digital media are only “new” technologies to a limited extent; their significance and that of their possible and necessary applications, however, has shifted significantly amid preventive measures in the context of the Covid-19 crisis. Even in the medium and long term, in the post-pandemic phase, models of distance learning and homeschooling will continue to be part of everyday school life (Nikolov et al. 2018).

Accordingly, *physical classrooms*, artifacts, and configurations are primarily aimed neither at infection control nor at open -teaching concepts or alternative teaching-learning arrangements (Röhl 2016), which have thus far had to be adapted and improvised in teaching scenarios. *Virtual classrooms* open up a wider and more extensive horizon of teaching practices, and digitally supported teaching and learning arrangements and the inclusion of digital media in the subject cultures, which up to now have mostly been optional, will become obligatory in the foreseeable future. At the same time, this transformation of teaching and learning also offers the opportunity and the necessity to reflect on the pedagogical framework and common didactic concepts in a digitally supported curriculum and to transfer them into teaching practice.

When talking about digitally supported curriculum making, this does not mean that the use of new media, in the sense of digitization as a technical infrastructure program for the subjects, is unconditional or one-sided. Rather, we are discussing the expansion of a digital culture that is already inherent in educational systems as such but that is experiencing significant development through the progress of technical possibilities in recent decades and whose potential for schools can unfold in and after the pandemic.

Digital culture is inherent in educational systems because digitality is not limited to hardware, software, or digital media (i.e., it is not necessarily linked to the use of notebooks, tablets, smartphones, online resources, or apps). Digital culture can be characterized by attitudes and practices whose roots go back a long way: *referentiality* is the method by which “individuals can inscribe themselves into cultural processes and constitute themselves as producers” (Stalder 2018, p. 58). Thus, all those practices of citing, assembling, and paraphrasing with which we refer to already available sources and other cultural artifacts transform them and transfer them into something new. Learning processes, especially those aimed at transferring what has been learned in order to create new knowledge, new practices and artifacts, are already such processes of referencing. The change in the key media (Honegger 2017) from material and analog to virtual and digital (i.e., from the medium of the printed text to that of the virtual text) catalyzes and dynamizes these practices, of which there are many examples. Hyperlink and hypertext procedures, but also the copyright discussions concerning intellectual property on the World Wide Web, bear witness to these changes; however, they are fundamentally already present in school and academic work.

This finding also applies to two other characteristics modeled by Felix Stalder (2018): *communality* and *algorithmicity*. Communities are “formed in a field of practice, characterized by informal yet structured exchange, focused on the generation of new ways of knowing and acting, and maintained through the reflexive interpretation of their own activity” (p. 84.). Cultural practices that generate meaning—as well as the practice of referencing itself—cannot be realized by a single actor; cultural meaning and its mediation take place only in a social space and communicative framework. The dimensions in which communication takes place today have nevertheless become more diverse and complex through virtual social spaces and techniques. The reduction of such complexity represents one of the central tasks that algorithms are supposed to fulfill—that is, instructions for “converting a given input into a desired output by means of a finite number of steps” (p. 104). Instructions for use and regulations, and ultimately any form of rules and regulations, are also algorithms. The new aspect of the paradigm shift known as the digitalization push is thus not a digital one per se, however it may be interpreted; instead, it is a transfer of already established practices into new technological and media environments, which is leading to a new culture in educational systems. The task of a digitally supported curriculum, analogous to that of an algorithm, would be to reduce such complexity and to structure dynamic information using digital techniques and technologies to implement educational standards for the 21st century.

In terms of curriculum making, digital culture thus denotes at least two things. First, it denotes the further development of the form and structure of the curriculum itself. Integrated into the progress of digital practices—and in view of constantly growing content, the dynamic availability of information, and the fundamental indeterminability of future technological development—such a curriculum must define and provide learning and educational goals as well as methods for achieving them. Second, this also means the implementation of content and didactic instruments in the sense of imparting digital and subject-related competences and the methodical use of digitally supported teaching-learning arrangements in interdisciplinary and cross-curricular teaching. In the context of the Covid-19 crisis, both lines of development, and the necessity of their cultivation, are in contrast. They influence the relationship between teaching, learning, and education, and suggest a shift in emphasis in curriculum making that affects content, social forms, and instructional methods.

One of the current challenges for lesson planning and implementation is that distance learning models and restricted classroom teaching are isolating students and teachers. In addition to all the other functions a class assumes in terms of socialization, psychological development, and education in general, its capacity as a cooperative learning space is eliminated in the medium term and must be replaced by procedures such as direct instruction in analogue or digital media. However, these procedures are by no means inappropriate or even outdated; on the contrary, direct instruction in particular is an essential building block for guiding independent and self-responsible learning (Brüning and Saum 2019). Rather, the challenge lies in the obvious universalization of traditional modes of teaching via new media, which, however, does not take into account the potential for an integrative redesign of cooperative forms of learning.

In accordance with the factors of referentiality, communality, and algorithmicity outlined here, an essential characteristic of digital culture is its fundamental openness: the information that can be accessed online as well as the virtual communication spaces are fundamentally immeasurable in their diversity and breadth and require a reduction in complexity. However, available knowledge and social interaction possibilities always exceed the control of curricula, as long as they seek to concretely define the contents and social forms of teaching. A possible shift in emphasis of the digitization of schools would be to develop more learner-oriented teaching scenarios for the school subjects—for example, in the sense of learning tasks and phenomenon -based learning, instead of knowledge transfer, which promotes independent research, selection, processing, and presentation of knowledge and learning products by learners.

This is not about a fundamental revolution in teaching and educational standards but rather about a cautious expansion of the structures and practices already established in the educational system. Accordingly, for teachers, this development did not mean adapting their own teaching styles but rather being able to draw on extant resources and experience—in other words, not doing everything differently but doing what one does in a different way.

The facilitation of distance learning and distance education via digital media is an obvious option and is, in principle, easy to improvise. However, the short-term challenges in implementing this concept also consist in actually exploiting the full potential of digitally supported teaching settings without a prior concept, in teachers and learners finding their way together through the wide range of available offers, and in familiarizing themselves with the functioning of hardware and software and new forms of teaching—in short, in building up a functioning repertoire of methods. Such an understanding of digitally supported teaching remains one-sided, however, if it reduces digitality to teaching methods and digital media to aids that optionally expand classroom teaching and the subject curriculum, without influencing the content and forms of teaching and learning (Pratt and Kovatcheva 2018).

In this sense, the most recent implementations of media literacy frameworks are primarily oriented toward changes in the media as an addendum to primary and secondary school curricula, which focus on CIL and CT education across all subjects. Both approaches to digital literacy complement the school curriculum and aim at the individual acquisition of transversal skills. On the one hand, however, the focus is on media competences and not necessarily on the specific use of digital media in the individual subject cultures. On the other hand, the competence frameworks—not least because of their inter- and transdisciplinary character and the dynamics of an unmanageable and rapidly growing number of digital technologies and applications they seek to adapt—function as orientation grids with a high degree of abstraction. They are not suitable as a framework curriculum or for the concrete planning and implementation of subject-related digital educational standards.

Based on the necessity for an educational response within the framework of the Covid-19 intervention strategies and the worldwide switch to forced distance learning and teaching, the framework of digital curricula could be discussed using the example of the internationally different concepts of curriculum making on the one hand and the digital literacy implemented in educational systems on the other. The gap we have identified in the international comparison based on the current crisis using the example of Luxembourg indicates the need for a digital framework curriculum. Such a curriculum would be more than a repository of teaching methods on digital media and learning apps and would pursue more goals than the teaching of media-related skills. Rather, it would be a foundation reflected in the respective cultures of the teaching subjects, which (a) forms a matrix for the subject-related transfer of teaching contents, methods, and also social forms into the change of leading media, and in doing so; (b) takes into account the characteristics of digital culture in terms of referentiality, communality, and algorithmicity; and (c) offers an orientation for teachers and learners. Finally, as this was one of the starting points for the reflections on a possible curriculum crisis response, a further development of the curriculum designed in this way aims to reduce the digital use divide (Senkbeil et al. 2019), as it is not only at the technical infrastructure but also at the long-term digital transformation of the teaching culture.

The Covid-19 crisis is thus not only a short-term challenge for all those involved in school education but also could mark a milestone in the development of digital teaching culture and media education. This is not because teachers are forced to integrate digital media into their lessons but because it will lead democratic societies to decide how they want to use digitalization after the pandemic has passed.
